# Re-Exploring the Ability of Common Docking Programs to Correctly Reproduce the Binding Modes of Non-Covalent Inhibitors of SARS-CoV-2 Protease M^pro^

**DOI:** 10.3390/ph15020180

**Published:** 2022-01-31

**Authors:** Davide Bassani, Matteo Pavan, Giovanni Bolcato, Mattia Sturlese, Stefano Moro

**Affiliations:** Molecular Modeling Section (MMS), Department of Pharmaceutical and Pharmacological Sciences, University of Padova, 35131 Padova, Italy; davide.bassani.1@studenti.unipd.it (D.B.); matteo.pavan.7@phd.unipd.it (M.P.); giovanni.bolcato.1@phd.unipd.it (G.B.); mattia.sturlese@unipd.it (M.S.)

**Keywords:** molecular docking, molecular dynamics, SARS-CoV-2, main protease, M^pro^, docking benchmark

## Abstract

In the latest few decades, molecular docking has imposed itself as one of the most used approaches for computational drug discovery. Several docking benchmarks have been published, comparing the performance of different algorithms in respect to a molecular target of interest, usually evaluating their ability in reproducing the experimental data, which, in most cases, comes from X-ray structures. In this study, we elucidated the variation of the performance of three docking algorithms, namely GOLD, Glide, and PLANTS, in replicating the coordinates of the crystallographic ligands of SARS-CoV-2 main protease (M^pro^). Through the comparison of the data coming from docking experiments and the values derived from the calculation of the solvent exposure of the crystallographic ligands, we highlighted the importance of this last variable for docking performance. Indeed, we underlined how an increase in the percentage of the ligand surface exposed to the solvent in a crystallographic complex makes it harder for the docking algorithms to reproduce its conformation. We further validated our hypothesis through molecular dynamics simulations, showing that the less stable protein–ligand complexes (in terms of root-mean-square deviation and root-mean-square fluctuation) tend to be derived from the cases in which the solvent exposure of the ligand in the starting system is higher.

## 1. Introduction

In the 1980s, with the first study provided by Kuntz et. al [1], the computational technique of molecular docking had its birth. The efficiency, speed, and robustness of this method make its presence a constant in every structure-based drug-discovery pipeline [2]. To give a brief explanation, molecular docking consists of a multistep computational process that aims to find the best conformation of a molecule to bind to another to form a stable complex [3]. In the field of medicinal chemistry, as is deductible, its main application is finding the best molecules to bind in a firm way to the desired target (a protein, a nucleic acid, etc.). The algorithm starts with the exploration of the conformations space of the ligands (exploiting the so-called “search algorithm”). The conformations (called “poses”) are then classified by a “scoring function”, which attributes a numeric value to the goodness of the interaction according to energetical and/or geometrical function.

After a series of iterations, the final conformations are presented from the program to the user and ranked by the internal scoring function [4].

In the last 30 years, many docking programs have been developed. Among them, GOLD [5] (a genetic docking algorithm developed by the Cambridge Crystallographic Data Center—CCDC), Glide [6] (a systematic docking program released under license by Schrödinger), AutoDock [7] (a non-commercial genetic algorithm from The Scripps Research Institute), AutoDock VINA [8] (created by the same organization and released for non-commercial use), and PLANTS [9] (an algorithm based on an “Ant Colony Optimization” method) have gained popularity.

The performance of molecular docking programs can be measured by evaluating their ability to reproduce the experimental structural data, such as the crystallographic coordinates of a ligand into its binding site [10]. This ability has been evaluated in several benchmarks [11,12] to rank the performance of different algorithms regarding a specific target, usually using as the key parameter the root-mean-square deviation (RMSD) between the coordinates of the different poses given by the program and the crystallographic ones.

The ability to reproduce a crystallographic conformation strongly relies on different factors. First, the geometrical characteristics of the binding site, such as extension and shape, play a very important role; it is known that the performance of the algorithms has been improved to dock molecules in “cavities” or “pockets”, rather than surfaces of proteins [13]. Second, the nature and the dimensions of the ligand are also crucial. Indeed, very small ligands may explore different places in a binding site, and the interactions that they can establish are usually few in number, reducing the “synergism” which could induce a molecule to keep a peculiar shape in a pocket [14]. On the other hand, even if drug-like molecules generally have higher conformational freedom, their dimensions force them to be oriented into a site in a more conserved way, so they have less roto-translational freedom.

In this study, we examined the ability of three docking programs characterized by diverse conformational sampling algorithms to efficiently reproduce the crystallographic pose of different ligands bound in different sites of a protein. To accomplish this task, a target in which several crystal structures were solved with the ligands located in different sites of the macromolecule itself was needed. To this scope, we considered a very recent and relevant target in the current pharmaceutical scenario, namely the SARS-CoV-2 main protease (M^pro^).

**Figure 1:**
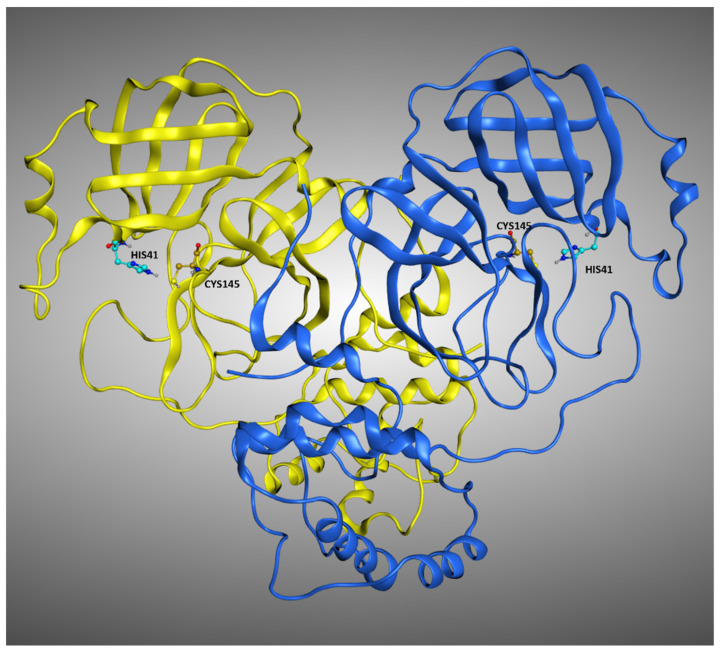
Representation of the crystal structure of M^pro^ (PDB:7L10). The two monomers composing the protein are colored differently, while the residues of the catalytic dyad, Cys145, and His41 are labeled in each of the monomers.

In the last couple of years, with the pandemic spreading of the SARS-CoV-2 virus, the world of medical sciences had found itself fighting a new and dangerous adversary [15,16]. This biological entity, which is part of the coronavirus family, has been demonstrated to cause a pulmonary infection which eventually leads to serious complications, as witnessed by the high number of deaths that have already been linked to it (more than 5 million, at the present day [17]). The replication cycle of this virus strongly relies on the activity of its main protease (known as M^pro^ or 3CL^pro^, a crystallographic structure example is reported in Figure 1) [18]. Indeed, this protein is responsible for the cleavage of the propeptide transcribed by the viral genome. In this way, the formation of all the functional proteins for the building of new virions takes place, and so the viral infection can proceed. Even if many molecules have been shown to bind to M^pro^ [19] and inhibit its activity, and even if a molecule is currently in phase III clinical trial for this purpose (PF-07321332, from Pfizer [20,21]), no drug has already been approved by the European Medicinal Agency for the treatment of SARS-CoV-2 (also called “COVID-19”). Computational methods have already proven to be beneficial in the research for new potential inhibitors for M^pro^, as the literature witnesses [22,23]. In this work, we decided to implement a molecular-docking-based approach relying on the programs GOLD, Glide, and PLANTS. These algorithms are considered “orthogonal” because they are characterized by diverse placing and scoring algorithms to obtain the best solution to the “protein–ligand posing problem”. Each of these programs was used to dock each of the different non-covalent ligands of the various crystal structures of M^pro^, and this allowed us to evaluate the factors which influence the variability in reproducing the crystallographic poses. A self-docking protocol similar to the one herein reported had already been developed by our laboratory, with the name “DockBench”. This program was implemented with success in several workflows, as the literature assesses [24,25]. In this study, a slightly modified version of that tool was used, which exploits only three docking programs at the present moment but can expand the analysis of the results obtained.

Looking at the docking benchmarking protocols on M^pro^, we see that a remarkable study has already been conducted and published by Zev et al. [26]. In that specific work, six different docking programs were evaluated in their performance in reproducing the M^pro^ non-covalent ligands’ crystallographic poses, and three of those algorithms have also been compared in their ability to correctly place M^pro^ covalent ligands into their proper binding site. In our work, we decided to expand the considerations brought by that study, evaluating specifically how docking performance changes in respect of the crystallographic data that have to be reproduced.

**Figure 2:**
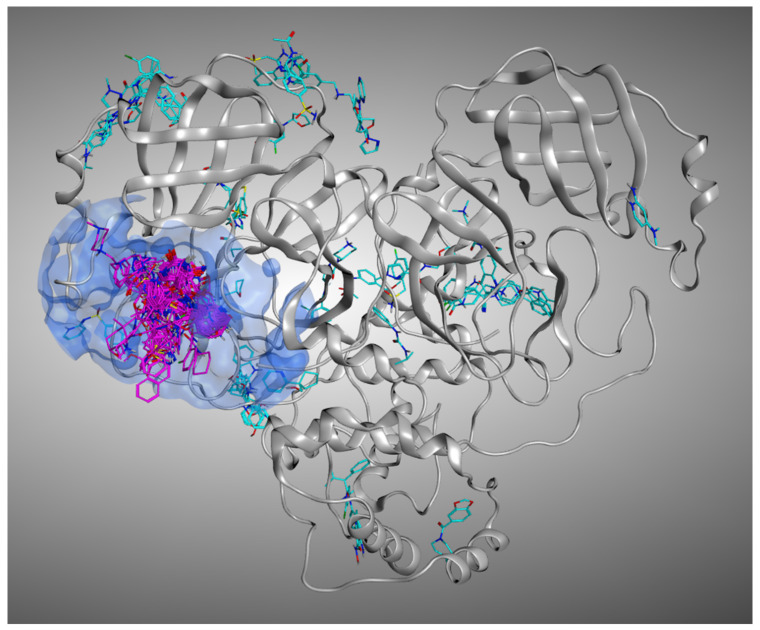
Representation of all the crystallographic ligands of M^pro^ superposed. To give a better view, just one protein structure is represented (the one coming from PDB:7L10). The ligands which are crystallized inside the catalytic pocket are colored in magenta, while all the small molecules crystallized outside the orthosteric binding site are colored in cyan.

Indeed, we considered in our calculations parameters such as the solvent exposure of the ligand and the influence of the crystallographic water molecules in docking calculations, focusing our evaluations just on non-covalent M^pro^ ligands. We executed the experiment in two different scenarios, one which excluded the crystallographic waters from the calculation (which we will name “Scenario 1”), and one which induced the docking programs to consider them (called “Scenario 2”). After that, we compared the docking results with the percentage of solvent exposure of the crystallographic pose of the ligand, successfully confirming that a higher solvent exposure tendentially reflects a worsening in the ability to reproduce the crystallographic pose by the algorithms (that, as already mentioned, are better trained for “cavities” rather than “surfaces”). To further investigate this aspect, we expanded our computational analysis by performing a molecular dynamics (MD) experiment, in which each crystallographic ligand was left free to move for 5ns (three replicas per system). This approach (known as “MD post-docking”) has already become part of our computational protocol [27,28] and is based on the fact that the conformations of the ligands, which are less prone to be displaced from their initial position during the simulation, are related to higher stability and binding strength with the target. In the case presented, this principle was applied directly to the crystallographic conformations of the ligands rather than to docking poses. This was performed because the goal was not to select the most promising molecules in binding to a specific region of the protein; instead, the objective was to elucidate which are the features of the crystallographic ligands that tend to guarantee a tighter binding with the receptor. Our evaluation demonstrated that the molecules bound to the orthosteric pocket of M^pro^ keep their position much stronger than the molecules crystallized on other sites, further validating our solvent exposure-based theory. A representation of the M^pro^ ligands crystallized in the various sites of the protein is given in Figure 2.

## 2. Results and Discussion

### 2.1. Scenario 1—Docking Calculations without Considering the Crystallographic Water Molecules

**Figure 3:**
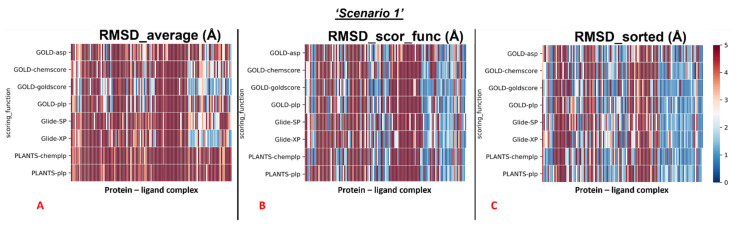
Colormaps represent the results of the self-docking experiments in the case in which the crystallographic water molecules are not considered during the docking runs. (**A**) Results coming from the average of the RMSDs of all the poses for each docking run. (**B**) Results derived just from the RMSD between the crystallographic ligand coordinates and the pose classified as the best from the scoring function. (**C**) Results of the self-docking experiments if just the pose showing the best RMSD value between its coordinates and the crystallographic ones are retained. The *x*-axis lists all the different protein–ligand complexes, which are plotted against the different pairs docking program-scoring function used for this study, reported in the *y*-axis.

The results of our docking protocol for this section (which are numerically reported in the Supplementary Materials File “Selfdocking_scenario1.csv”) are graphically represented with colormaps. All the colormaps present in this study are based on a colorimetric scale delineating the RMSD values, starting from 0 Å, which corresponds to a molecular docking pose exactly super posable to the crystallographic one (maximum docking performance, represented by the dark blue color), and reaching values of 5 Å or higher (minimum docking performance, all represented by the dark red color), corresponding to a very low level of overlap between the coordinates of the pose produced and the ones of the crystallographic conformation. The colormaps in Figure 3 show the self-docking results obtained on the different M^pro^ crystal structures in the case in which water molecules are not considered in the calculation. As is depicted, the RMSD values were far lower for all the complexes in which the crystallographic ligand is located in the orthosteric pocket.

To give a better resolution of this, we separated each map into two different colormaps, one grouping all the 78 proteins in which the ligand is located into the catalytic pocket, and one including all other cases (41 complexes).

We analyzed the data coming from the calculations, and we determined that, looking at all the complexes with all the different couples docking program-scoring functions, we see that the average values of all the RMSDs obtained was 5.76Å (“RMSD_average”). Looking at the average of the RMSDs coming from the poses which were scored as the best ones from the algorithms’ scoring functions (“RMSD_scor_func”), we see that the value was 5.10Å. If the lowest RMSD values only are taken into account for each docking run (“RMSD_sorted”), the mean of the values was 3.70Å.

**Figure 4:**
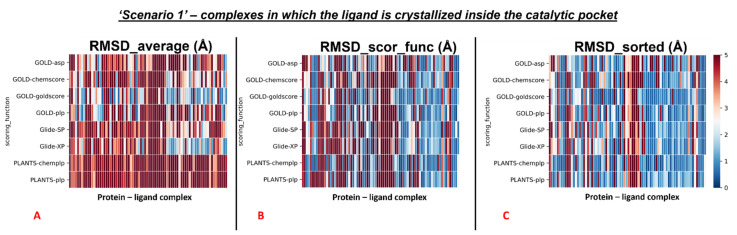
Colormaps represent the results of the self-docking experiments just for the ligands crystallized inside the orthosteric pocket in the situation in which the crystallographic water molecules are not considered during the docking runs. (**A**) Results coming from the average of the RMSDs of all the poses for each docking run. (**B**) Results derived just from the RMSD between the crystallographic ligand coordinates and the pose classified as the best from the scoring function. (**C**) Results of the self-docking experiments if just the pose showing the best RMSD value between its coordinates and the crystallographic ones is retained. The *x*-axis lists all the different protein–ligand complexes, which are plotted against the different pairs docking program-scoring function used for this study, reported in the *y*-axis.

**Figure 5:**
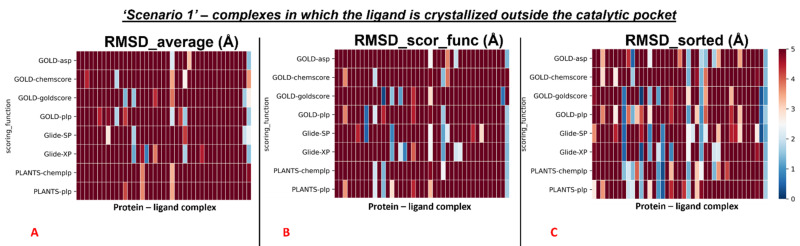
Colormaps represent the results of the self-docking experiments just for the ligands crystallized outside the orthosteric pocket in the case in which the crystallographic water molecules are not considered during the docking runs. (**A**) Results coming from the average of the RMSDs of all the poses for each docking run. (**B**) Results derived just from the RMSD between the crystallographic ligand coordinates and the pose classified as the best from the scoring function. (**C**) Results of the self-docking experiments if just the pose showing the best RMSD value between its coordinates and the crystallographic ones is retained. The *x*-axis lists all the different protein–ligand complexes, which are plotted against the different pairs docking program-scoring function used for this study, reported in the *y*-axis.

The average values were also calculated separately for all the complexes in which the crystallographic ligand is located in the catalytic pocket, and for all other cases. The colormaps for these different conditions are reported in Figure 4 and Figure 5.

First, the analysis focused on the complexes having the crystallographic ligand located within the orthosteric pocket. For this set of systems, we calculated the average RMSD value of all the poses (“RMSD_average”), which was revealed to be 4.54Å. Then we computed the average of the RMSD values coming from the poses which were ranked with the best score from the scoring functions (“RMSD_scor_func”), and its value was 3.43Å. Finally, the average RMSD value of the poses with the lowest RMSD in each run was calculated (“RMSD_sorted”), and its measure was 2.45Å.

Second, the same steps were performed for the rest of the protein–ligand complexes, which are the ones in which the crystallographic ligand is located outside the orthosteric binding site. Moreover, in this case, the first passage involved the calculation of the average RMSD value of all the poses generated for these systems (“RMSD_average”), and its measure was 8.08Å. Then, the mean of the RMSD values coming from the poses which received the highest rank from the scoring functions was calculated (“RMSD_scor_func”) and was revealed to be 8.29Å. In the end, the average value of the lowest RMSDs of each run was computed (“RMSD_sorted”), and its measure was shown to be 6.08Å.

The results obtained for Scenario 1 are summarized in Table 1.

### 2.2. Scenario 2—Docking Calculations Considering the Crystallographic Water Molecules

**Figure 6:**
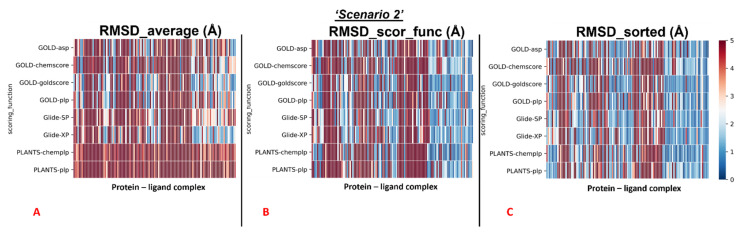
Colormaps represent the results of the self-docking experiments in the case in which the crystallographic water molecules at 5 Å or nearer to the ligand itself are taken into account during the docking runs. (**A**) Results coming from the average of the RMSDs of all the poses for each docking run. (**B**) Results derived just from the RMSD between the crystallographic ligand coordinates and the pose classified as the best from the scoring function.(**C**)Results of the self-docking experiments if just the pose showing the best RMSD value between its coordinates and the crystallographic ones is retained. The *x*-axis lists all the different protein–ligand complexes, which are plotted against the different pairs docking program-scoring function used for this study, reported in the *y*-axis.

**Figure 7:**
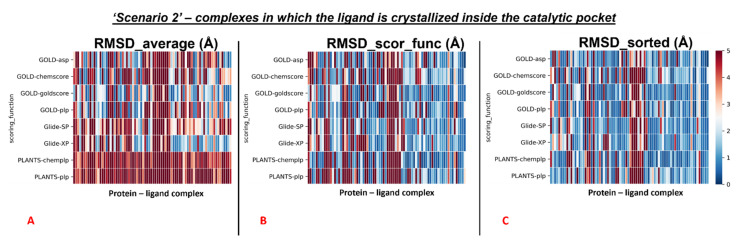
Colormaps represent the results of the self-docking experiments just for the ligands crystallized inside the orthosteric pocket in the situation in which the crystallographic water molecules at 5 Å or nearer to the ligand itself are taken into account during the docking runs. (**A**) Results coming from the average of the RMSDs of all the poses for each docking run. (**B**) Results derived just from the RMSD between the crystallographic ligand coordinates and the once of the pose classified as the best from the scoring function. (**C**) Results of the self-docking experiments if just the pose showing the best RMSD value between its coordinates and the crystallographic ones are retained. The *x*-axis lists all the different protein–ligand complexes, which are plotted against the different pairs docking program-scoring function used for this study, reported in the *y*-axis.

**Figure 8:**
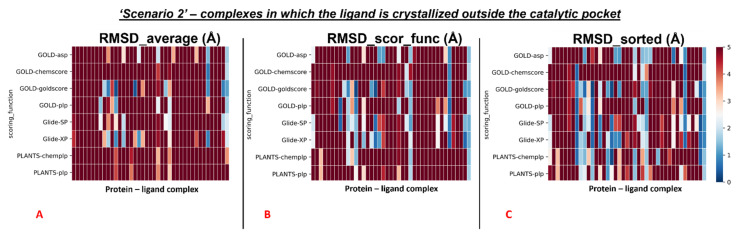
Colormaps represent the results of the self-docking experiments only for the ligands crystallized outside the orthosteric pocket in the situation in which the crystallographic water molecules at 5 Å or nearer to the ligand itself are taken into account during the docking runs. (**A**) Results coming from the average of the RMSDs of all the poses for each docking run. (**B**) Results derived just from the RMSD between the crystallographic ligand coordinates and the once of the pose classified as the best from the scoring function. (**C**) Results of the self-docking experiments if just the pose showing the best RMSD value between its coordinates and the crystallographic ones are retained. The *x*-axis lists all the different protein–ligand complexes, which are plotted against the different pairs docking program-scoring function used for this study, reported in the *y*-axis.

The outcomes of our molecular docking experiment for this section (which are reported in the Supplementary Materials File “Selfdocking_scenario2.csv”) are graphically represented with colormaps, which were created with the same criteria listed in the previous paragraph. The results reported in the colormaps in Figure 6, Figure 7 and Figure 8 reveal the self-docking performance obtained on the different M^pro^ crystal structures in the case in which the crystallographic water molecules within 5 Å from the ligand were retained during the calculation. Moreover, in this case, it is easy to notice that the values result in being far better for the complexes in which the small molecule of interest is in the orthosteric binding site.

Similar to the first scenario, we divided each colormap into two sets, one with the 78 proteins having the ligand located into the catalytic pocket, and the other including all the remaining cases (41 proteins). Considering all the protein–ligand complexes with all the different pairs docking program-scoring function, the mean values of all the RMSDs obtained (“RMSD_average”) was 5.64Å, but focusing only on the mean of the RMSDs derived from the poses which were given the highest rank from the algorithms (“RMSD_scor_func”), the value resulted to be 4.83Å. Looking only at the best RMSDs for each docking run (“RMSD_sorted”), we see that the average of the values was 3.68 Å.

As already performed for Scenario 1, also in Scenario 2, the analysis was divided between the complexes in having the crystallographic ligand crystallized into the catalytic pocket, and for all other situations.

We reported the colormaps which resulted from this evaluation, and those are represented in Figure 7 and Figure 8.

We started from the complexes in which the ligand is located inside the catalytic pocket in the crystal. For those systems, the mean of the RMSD values coming from all the poses(“RMSD_average”) resulted in being 4.22Å. Then, the average of the RMSDs derived from the scoring function highest-ranked poses in all the docking runs (“RMSD_scor_func”) was computed, and its value was 3.11Å. In the end, also the average value between the lowest of the RMSDs in each docking run was calculated (“RMSD_sorted”) and was revealed to be 2.26Å.

Second, we repeated the analysis for all the complexes in which the crystallographic ligand is located outside the orthosteric pocket. For these systems, the average of the RMSD coming from all the poses collected in the docking runs (“RMSD_average”) was calculated to be 8.32Å. Next, we computed the mean of the RMSD values derived from the poses which received the highest score (from the scoring functions) in each run (“RMSD_scor_func”), and this value was 8.11Å. Last, also the average value between the lowest of the RMSDs in each docking run was calculated (“RMSD_sorted”), giving 6.36 Å.

The results obtained for Scenario 1 are summarized in Table 2.

Just analyzing the numbers coming from the average values allows us to see how the performance of the docking programs dramatically increases when the ligand is docked inside the catalytic pocket rather than on the surface of the protein, in line with the fact that the molecules have a limitation in the conformation that they can explore into a binding site. Together with this, the ligands can exploit their accessible surface area to interact with the protein more efficiently, following the principle of “complementarity” [29,30].

### 2.3. Solvent Exposure Analysis

**Figure 9:**
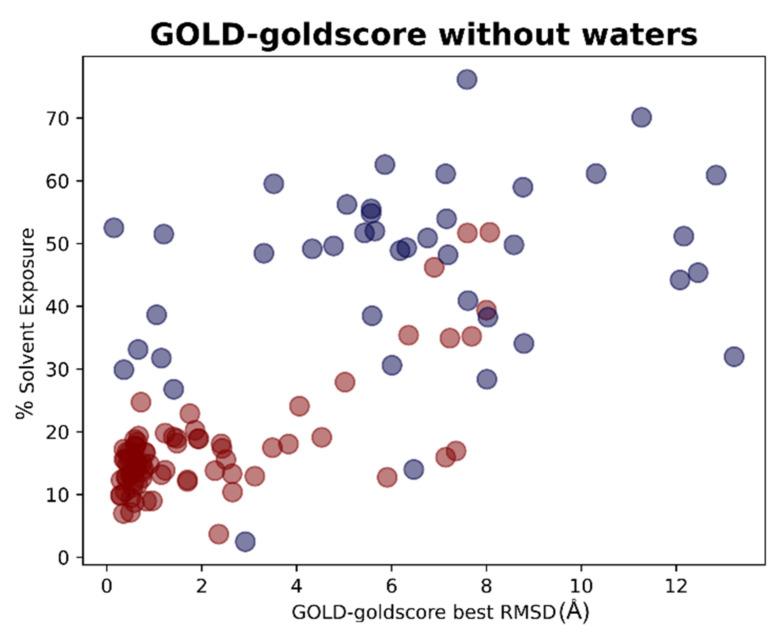
Scatter plots showing the different distribution of the RMSD values between the coordinates of the best pose from the GOLD-goldscore docking experiment in respect to the solvent exposure of the corresponding crystallographic ligands. The red dots represent the values having the ligand crystallized inside the catalytic pocket while the blue dots represent the ligands crystallized on the other sites of M^pro^. As can be noticed, the molecules showing the best values of RMSD are, in most cases, located inside the orthosteric pocket and characterized by low solvent exposure. This plot depicts part of the results of Scenario 1, and so the crystallographic water molecules are not considered in the docking runs of which the outcomes are here represented.

**Figure 10:**
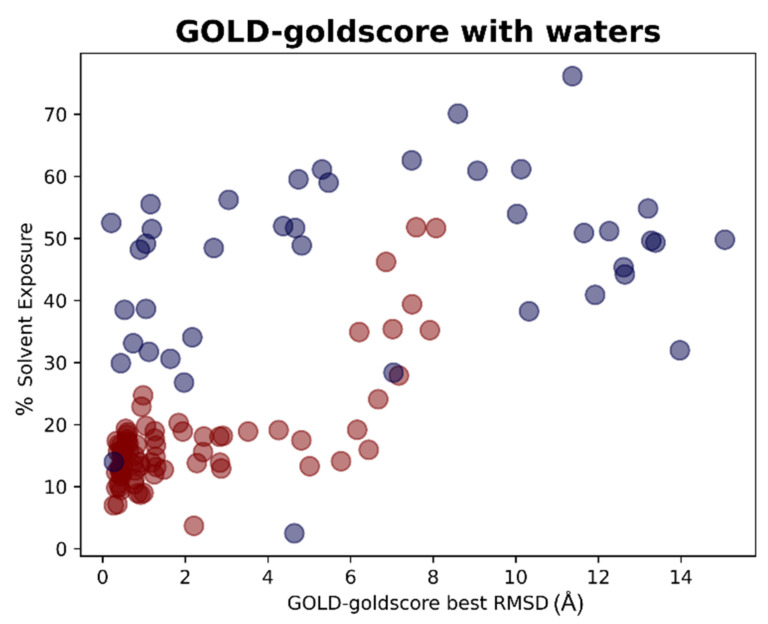
Scatter plots showing the different distribution of the RMSD values between the coordinates of the best pose from the GOLD-goldscore docking experiment in respect to the solvent exposure of the corresponding crystallographic ligands. The red dots represent the ligands that are originally crystallized inside the catalytic pocket, while the blue dots represent the ligands crystallized in the other parts of M^pro^. As can be noticed, the molecules showing the best values of RMSD are in most cases located inside the orthosteric pocket and characterized by low solvent exposure. This plot depicts part of the results of Scenario 2, meaning that the crystallographic water molecules at 5 Å or nearer to the ligand are also considered in the docking runs of which the outcomes are here represented.

The results of the docking calculations were then analyzed in light of the data coming from the solvent exposure analysis. For each docking program-scoring function pair, the best RMSDs given by the docking calculation were evaluated against the solvent exposure of the ligand in its crystallographic pose. The results were reported in different plots, one for each couple docking program-scoring function, also in this case dividing the graphs in respect to the “scenario” from which the docking result was coming. To give an example, we reported in this article the plots for the pair GOLD-goldscore for each of these cases (Figure 9 and Figure 10).

**Figure 11:**
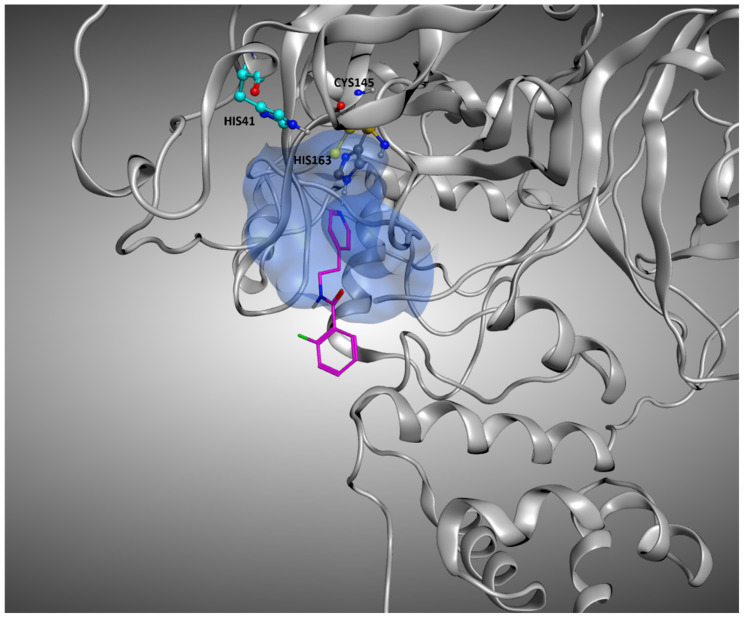
Representation of the crystallographic complex conformation of 5RGK, one of the protein–ligand complexes in which the crystallographic ligand is located inside the orthosteric binding site, but the docking calculation results in high RMSD values. This is mainly due to the high level of solvent exposure that characterizes this ligand, which locates just a small portion of its structure inside the pocket, leaving the rest in an outer zone. The ligand is represented with stick representation (C-atom are colored in magenta), and the catalytic dyad (Cys145 and His41) is highlighted, as well as the His163 and the binding site residue interacting with the ligand. To give a better representation, the surface of the protein in a 5 Å radius from the ligand is represented and colored in blue.

The plots arising from all other docking program-scoring function pairs, both in Scenario 1 and Scenario 2, are reported in Supplementary Materials Figure S1. From these graphs, we can easily see how the best RMSDs values tended to be derived from protein–ligand complexes in which the solvent exposure of the ligand is low, and, most of the time, this means that the small molecule is crystallized in the binding pocket (indicated with the red dots in the plots). There are some cases in which the mean RMSD values were suboptimal also for this kind of ligands, and this can be due to several reasons. In some situations, of which the complexes 5REH, 5RE9, 5RGK (represented in Figure 11), and 7AVD are an example, the solvent exposure was tendentially higher in respect to the other orthosteric ligands, while, in other cases, the increase in RMSD can be attributable to the small dimensions of the ligand itself, making it harder for the docking algorithms to reproduce the reference pose in a pocket of such considerable volume (the complexes 5R82 and 5RG0 are an example for this) [31].

**Figure 12:**
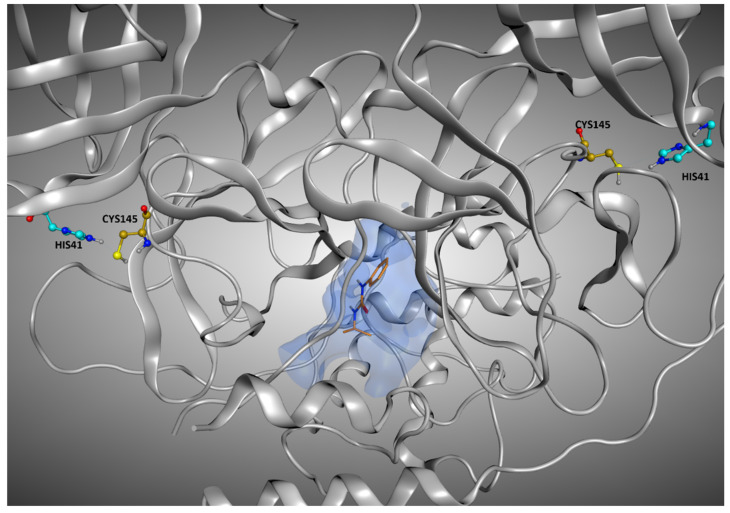
Representation of the crystallographic pose of 7LFP, which is one of the protein–ligand complexes in which, even if the crystallographic ligand is located outside the orthosteric binding site, the RMSD values between the original coordinates and the ones given from the docking runs are considerably low. The reason for this can be found in the very low solvent exposure of this ligand, which is located in the interface between the monomers, and so is shielded by them. The ligand is represented in orange, and the catalytic dyad (Cys145 and His41) of both monomers is highlighted. To give a better representation, the surface of the protein in a 5 Å radius from the ligand is represented and colored in blue.

On the other hand, there are also some cases in which the best RMSD given by the protocol was pretty low, even if the crystallographic ligand was not placed inside the orthosteric pocket. This is the case, for example, of 7LFP (the crystallographic pose is reported in Figure 12); the ligand was placed at the interface between the monomers, and so its solvent exposure and RMSDs values were low, even if was marked to be “outside the catalytic pocket”. A similar situation is observed on 5RF0, where the ligand, even if not located into the orthosteric pocket, is not situated in the peripheral part of the protease.

### 2.4. Molecular Dynamics Simulations

For each of the 119 crystallographic complexes, three different molecular dynamics simulations (MD) of 5 ns each were collected to examine the behavior of the ligands in a dynamic environment. The trajectories were wrapped, aligned to the first frame and the root-mean-square fluctuation (RMSF) of the ligand, as well as the RMSD between its crystallographic and final coordinates (“RMSD_final”), and were calculated for every single experiment. For each protein, the values coming from the average of the RMSFs and “RMSD_final” derived from the replicas were considered. Considering all the simulations collected, the average of all the ligand RMSF values was calculated to be 5.28 Å, while the average of the RMSD values between the coordinates of the crystallographic conformation of the ligand and the ones coming from the last frame of the trajectory (“RMSD_final”) was of 8.89 Å.

As already performed for the docking results analysis, we first focused on the complexes in which the crystallographic ligand is originally located inside the orthosteric pocket. For these systems, the average of all the RMSFs coming from the simulations was 2.19 Å. The mean value of the RMSDs of the ligands in the last frame of each trajectory (“RMSD_final”) was instead calculated to be 4.43 Å.

Second, we concentrated on the systems in which the crystallographic position of the ligand (and so its initial location) is outside the catalytic pocket. For these systems, the average value of all the ligand RMSFs during the trajectories was calculated to be 11.15Å. Then the RMSD value between the final coordinates of the ligands and their crystallographic ones (“RMSD_final”) were considered. The average of these values, for all the trajectories collected for these complexes, was 17.66Å. The output files of the molecular dynamics simulation geometric analysis are available in Supplementary Materials “MD_data.csv”.

**Figure 13:**
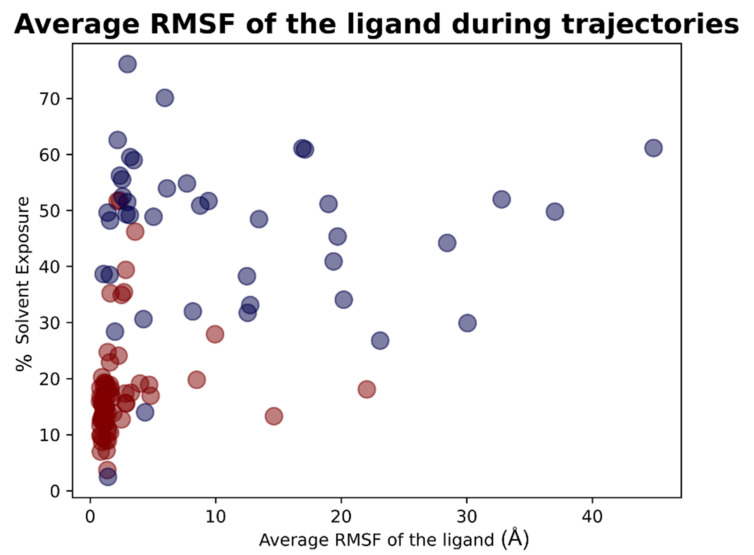
Scatter plots showing the different distribution of the mean RMSF values between the coordinates of the M^pro^ ligands compared to crystallographic ones after the molecular dynamics simulations in respect to the solvent exposure of the corresponding crystallographic ligands. The red dots represent the ligands thatwere originally crystallized inside the catalytic pocket, while the blue dots represent the ligands crystallized in the other parts of M^pro^. As can be noticed, the molecules showing the best values of RMSF after the analysis of the trajectories are mainly located inside the catalytic pocket and characterized by a low solvent exposure of the original crystallographic pose.

**Figure 14:**
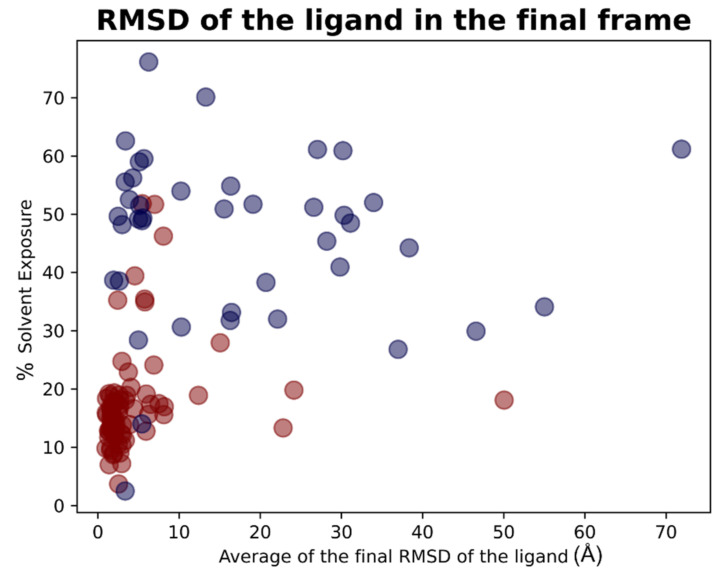
Scatter plots showing the different distribution of the mean RMSD values between the final coordinates of the M^pro^ ligands compared to crystallographic ones after the molecular dynamics simulations in respect to the solvent exposure of the corresponding crystallographic ligands. The red dots represent the ligands thatwere originally crystallized inside the catalytic pocket, while the blue dots represent the ligands crystallized in the other parts of M^pro^. As can be noticed, the molecules showing the best values of RMSF after the analysis of the trajectories are mainly located inside the catalytic pocket and characterized by a low solvent exposure of the original crystallographic pose.

As already performed for the docking experiments, also for MD results, the average values of RMSF and “RMSD_final” were plotted against the percentage of solvent exposure of the crystallographic conformation of the ligand, and the plots that were obtained are reported in Figure 13 and Figure 14.

As expected, the complexes in which the ligand is crystallized in the orthosteric site (marked with the red dots in the scatter plot) tended to fluctuate much less than the ligands which are complexed in the external parts of the protease (represented with the blue dots in the graphs). As depicted, MD analysis confirms that the best values in terms of RMSF and “RMSD_final”, which are correlated to a more energetically stable situation for the protein–ligand complex, come from the systems in which the crystallographic ligand is localized inside the catalytic pocket and are characterized by a low percentage of solvent exposure. These outcomes further support the already-mentioned hypothesis about the correlation between the improvement of the docking performances in the case in which the binding site is a pocket rather than a surface.

The overall results obtained with molecular dynamics simulations are summarized in Table 3. A graphical representation of the molecular dynamics simulations is reported in Supplementary Materials “Video_S1.mp4”. In this video, the ligands crystallized into the catalytic pocket are colored in magenta, while the other ligands are colored in cyan.

## 3. Materials and Methods

### 3.1. Software Overview

The molecular modeling operations were executed with the Molecular Operating Environment (MOE) suite (version 2019.01) [32]. The molecular docking calculations were carried out with CCDC GOLD (version 2020), Schrodinger Glide (from the Schrödinger suite 2021.3), and PLANTS. The solvent exposure calculation was performed with a series of SVL commands (exploiting “moebatch” of the MOE suite) implemented into a python script. The systems for molecular dynamics simulations were prepared by using tleap [33] and VMD [34]. The simulations were then carried out with ACEMD3 [35](version 3.3.0), a licensed program based upon OpenMM [36] (version 7.4.0). The modeling and docking calculations were performed on a 12 CPU (Intel Xeon E5-1620 3.50 GHz) Linux Workstation, while the MD simulations were carried out on a GPUs-cluster based composed of 20 NVIDIA GPUs.

### 3.2. Structure Preparation for Docking Calculations

The different crystal structures of M^pro^ were collected from the Protein Data Bank [37]. Among these, the proteins which did not present any ligand, or which were complexed with a covalent ligand, were excluded. This way, only the non-covalent protein–ligand complexes were retained, and the complete list of all the 119 complexes used is available in Supplementary Material Table S1. The structures were grouped into a database and were prepared with MOE “QuickPrep” tool. With this tool, each complex was properly prepared to recreate the small missing loops in the structure, assigning the proper conformation to the residues with alternate orientation (based on occupancy) and adding the hydrogens to the system (this last passage was performed with the MOE “Protonate 3D” tool). The hydrogens added this way were then minimized by using the AMBER10:EHT force field implemented in MOE [38].

After these preliminary but crucial steps, each complex was manually examined and treated to eliminate every molecule, except for the crystallographic waters and the main ligand. Each complex was then independently saved.

### 3.3. Docking Calculations

For each of the complexes prepared, the crystallographic ligand was separated from the protein and self-docked into its binding site. For each docking program, all the scoring functions available were used for separate runs, and in each run, 5 poses were collected for the ligand. GOLD supports 4 different scoring functions: goldscore, chemscore, asp, and plp; Glide supports two main functions for docking, which are Glide-SP and Glide-XP, while PLANTS implements plp and chemplp.

For each docking-program-scoring function couple, the docking calculation was carried out in two different scenarios: one in which the crystallographic water molecules were not considered (which we refer to as Scenario 1) and one in which also the water molecules 5 Å or nearer from the ligand atoms were taken into account into the computation (which we refer to as Scenario 2).

When all the docking calculations were executed, the ligand root-mean-square deviation (RMSD) between the coordinates of each one of the poses and the crystallographic conformations were computed. The data of major interest were the RMSD in respect to the pose which is marked with the highest score by the program (RMSD_scor_func), the lowest RMSD of the docking run (RMSD_sorted), and the average of the RMSDs of all the poses generated (RMSD_average). The output files of the self-docking experiments executed are available in the Supplementary Materials (“Selfdocking_scenario1.csv” and “Selfdocking_scenario2.csv”).

### 3.4. Solvent Exposure Calculation

For each M^pro^ complex, the solvent exposure of the main crystallographic ligand was calculated with an SVL script based on MOE “moebatch”. The output of such calculation was the percentage of the ligand surface which is exposed to the solvent in the protein–ligand crystallographic complex. All the percentages obtained are presented in Supplementary Materials Table S2.

### 3.5. Molecular Dynamics Simulations Setup and Execution

All the protein–ligand M^pro^ systems were independently prepared for molecular dynamics simulations. The program tleap was used for the creation of the simulation box, which was set to be cubic and characterized by a 15 Å padding. The solvation model used was the explicit TIP3P, and the neutralization of the system was performed by adding Na^+^ and Cl^−^ ions until the salt concentration inside the box reached the value of 0.154 M.

The systems then underwent a two-passage equilibration. In the first one, both protein and ligand atoms were subjected to a harmonic position restrain of 5 kcal/mol. The length of this step was set to 0.1 ns, and the ensemble used was the canonical one (NVT). During the second equilibration step, the ensemble was set to NPT (isothermal–isobaric), the length was 0.5 ns, and the harmonic restrains (always 5 kcal/mol) were applied both on the ligand and on the alpha-carbons of the protein backbone.

After these preliminary steps, 3 replicas of 5 ns each were collected for each system; the ensemble was again the NVT one, and no restraints were applied. At the end of the simulations, the average root-mean-square fluctuation (RMSF) of the ligand during the trajectory, as well as the RMSD betweencrystallographic coordinates of the ligand and the ones coming from the last frame of the trajectory, were collected.

## 4. Conclusions

In this study, we evaluated the performance of three orthogonal docking algorithms in reproducing the crystallographic pose of several ligands located in different parts of the same target, which, in our case, was the SARS-CoV-2 main protease (M^pro^). Our analysis revealed how, even if the docking programs used operate in different ways to give the final conformations to the user, all of them perform much better in the case in which the ligands are located in a binding pocket rather than crystallized outside of it. Specifically, we reported that their performance tends to decrease with the increment of the exposure of the crystallographic pose to the solvent. This was confirmed both from the experiments executed without considering the crystallographic water molecules in the docking calculations and from the ones taking into account the waters 5 Å or nearer to the ligand. Molecular dynamics simulations further give credit to our study, demonstrating how the less-fluctuating ligands (and so the most stable) through the trajectories were the ones crystallized inside the orthosteric binding site of M^pro^.

## Figures and Tables

**Table 1:** Table representing the self-docking results obtained for Scenario 1.

Results for Scenario 1—Docking Calculations Executed without Considering the Water Molecules			
	RMSD_Average (Å)	RMSD_Scor_Func (Å)	RMSD_Sorted (Å)
All the 119 protein–ligand complexes	5.76	5.10	3.70
The 78 complexes with the ligand inside the catalytic pocket	4.54	3.43	2.45
The 41 complexes with the ligand outside the catalytic pocket	8.08	8.29	6.08

**Table 2:** Table representing the self-docking results obtained for Scenario 2.

Results for Scenario 2—Water Molecules 5 Å or Nearer to the Ligand Considered in Docking Calculations			
	RMSD_Average (Å)	RMSD_Scor_Func (Å)	RMSD_Sorted (Å)
All the 119 protein–ligandcomplexes	5.64	4.83	3.68
The 78 complexes with the ligand inside the catalytic pocket	4.22	3.11	2.26
The 41 complexes with the ligand outside the catalytic pocket	8.32	8.11	6.36

**Table 3:** Results of the molecular dynamics experiments.

Results of the Molecular Dynamics Simulations		
	RMSD_Final (Å)	RMSF_Average (Å)
All the 119 protein–ligandcomplexes	8.98	5.28
The 78 complexes with the ligand inside the catalytic pocket	4.43	2.19
The 41 complexes with the ligand outside the catalytic pocket	17.66	11.15

## Data Availability

Data are contained within the article and Supplementary Materials.

## Supplementary Materials

The following are available online at https://www.mdpi.com/article/10.3390/ph15020180/s1: “Supplementary_material.docx” (containing Table S1, Table S2, and Figure S1), the CSV files “Selfdocking_scenario1.csv”, “Selfdocking_scenario2.csv” and “MD_data.csv”, “Video_S1.mp4”.

## References

[1] Kuntz I.D., Blaney J.M., Oatley S.J., Langridge R., Ferrin T.E. (1982). A geometric approach to macromolecule-ligand interactions. J. Mol. Biol..

[2] Meng X.-Y., Zhang H.-X., Mezei M., Cui M. (2011). Molecular Docking: A Powerful Approach for Structure-Based Drug Discovery. Curr. Comput. Aided-Drug Des..

[3] Lengauer T., Rarey M. (1996). Computational methods for biomolecular docking. Curr. Opin. Struct. Biol..

[4] Kitchen D.B., Decornez H., Furr J.R., Bajorath J. (2004). Docking and scoring in virtual screening for drug discovery: Methods and applications. Nat. Rev. Drug Discov..

[5] Jones G., Willett P., Glen R.C., Leach A.R., Taylor R. (1997). Development and validation of a genetic algorithm for flexible docking. J. Mol. Biol..

[6] Halgren T.A., Murphy R.B., Friesner R.A., Beard H.S., Frye L.L., Pollard W.T., Banks J.L. (2004). Glide: A New Approach for Rapid, Accurate Docking and Scoring. 2. Enrichment Factors in Database Screening. J. Med. Chem..

[7] Morris G.M., Huey R., Lindstrom W., Sanner M.F., Belew R.K., Goodsell D.S., Olson A.J. (2009). AutoDock4 and AutoDockTools4: Automated docking with selective receptor flexibility. J. Comput. Chem..

[8] Trott O., Olson A.J. (2009). AutoDock Vina: Improving the speed and accuracy of docking with a new scoring function, efficient optimization, and multithreading. J. Comput. Chem..

[9] Korb O., Stützle T., Exner T.E. PLANTS: Application of Ant Colony Optimization to Structure-Based Drug Design. Proceedings of the ANTS: International Workshop on Ant Colony Optimization and Swarm Intelligence.

[10] Yusuf D., Davis A.M., Kleywegt G.J., Schmitt S. (2008). An Alternative Method for the Evaluation of Docking Performance: RSR vs RMSD. J. Chem. Inf. Model..

[11] Boittier E.D., Tang Y.Y., Buckley M.E., Schuurs Z.P., Richard D.J., Gandhi N.S. (2020). Assessing Molecular Docking Tools to Guide Targeted Drug Discovery of CD38 Inhibitors. Int. J. Mol. Sci..

[12] Wang Z., Sun H., Yao X., Li D., Xu L., Li Y., Tian S., Hou T. (2016). Comprehensive evaluation of ten docking programs on a diverse set of protein–ligand complexes: The prediction accuracy of sampling power and scoring power. Phys. Chem. Chem. Phys..

[13] Ramirez U.D., Myachina F., Stith L., Jaffe E.K. (2010). Docking to Large Allosteric Binding Sites on Protein Surfaces. Advances in Experimental Medicine and Biology.

[14] Jacquemard C., Drwal M.N., Desaphy J., Kellenberger E. (2019). Binding mode information improves fragment docking. J. Cheminform..

[15] Fan Y., Zhao K., Shi Z.-L., Zhou P. (2019). Bat Coronaviruses in China. Viruses.

[16] Lotfi M., Hamblin M.R., Rezaei N. (2020). COVID-19: Transmission, prevention, and potential therapeutic opportunities. Clin. Chim. Acta.

[17] World Health Organization (WHO) WHO Coronavirus (COVID-19) Dashboard. https://covid19.who.int/.

[18] Jin Z., Du X., Xu Y., Deng Y., Liu M., Zhao Y., Zhang B., Li X., Zhang L., Peng C. (2020). Structure of Mpro from SARS-CoV-2 and discovery of its inhibitors. Nature.

[19] Zhang C.-H., Stone E.A., Deshmukh M., Ippolito J.A., Ghahremanpour M.M., Tirado-Rives J., Spasov K.A., Zhang S., Takeo Y., Kudalkar S.N. (2021). Potent Noncovalent Inhibitors of the Main Protease of SARS-CoV-2 from Molecular Sculpting of the Drug Perampanel Guided by Free Energy Perturbation Calculations. ACS Central Sci..

[20] Owen D.R., Allerton C.M.N., Anderson A.S., Aschenbrenner L., Avery M., Berritt S., Boras B., Cardin R.D., Carlo A., Coffman K.J. (2021). An oral SARS-CoV-2 M pro inhibitor clinical candidate for the treatment of COVID-19. Science.

[21] Pavan M., Bolcato G., Bassani D., Sturlese M., Moro S. (2021). Supervised Molecular Dynamics (SuMD) Insights into the mechanism of action of SARS-CoV-2 main protease inhibitor PF-07321332. J. Enzym. Inhib. Med. Chem..

[22] Rudrapal M., Celik I., Khan J., Ansari M.A., Alomary M.N., Yadav R., Sharma T., Tallei T.E., Pasala P.K., Sahoo R.K. (2022). Identification of bioactive molecules from Triphala (Ayurvedic herbal formulation) as potential inhibitors of SARS-CoV-2 main protease (Mpro) through computational investigations. J. King Saud Univ. Sci..

[23] Di Sarno V., Lauro G., Musella S., Ciaglia T., Vestuto V., Sala M., Scala M.C., Smaldone G., Di Matteo F., Novi S. (2021). Identification of a dual acting SARS-CoV-2 proteases inhibitor through in silico design and step-by-step biological characterization. Eur. J. Med. Chem..

[24] Cuzzolin A., Sturlese M., Malvacio I., Ciancetta A., Moro S. (2015). DockBench: An Integrated Informatic Platform Bridging the Gap between the Robust Validation of Docking Protocols and Virtual Screening Simulations. Molecules.

[25] Bolcato G., Cuzzolin A., Bissaro M., Moro S., Sturlese M. (2019). Can We Still Trust Docking Results? An Extension of the Applicability of DockBench on PDBbind Database. Int. J. Mol. Sci..

[26] Zev S., Raz K., Schwartz R., Tarabeh R., Gupta P.K., Major D.T. (2021). Benchmarking the Ability of Common Docking Programs to Correctly Reproduce and Score Binding Modes in SARS-CoV-2 Protease Mpro. J. Chem. Inf. Model..

[27] Bolcato G., Bissaro M., Sturlese M., Moro S. (2020). Comparing Fragment Binding Poses Prediction Using HSP90 as a Key Study: When Bound Water Makes the Difference. Molecules.

[28] Bolcato G., Cescon E., Pavan M., Bissaro M., Bassani D., Federico S., Spalluto G., Sturlese M., Moro S. (2021). A Computational Workflow for the Identification of Novel Fragments Acting as Inhibitors of the Activity of Protein Kinase CK1δ. Int. J. Mol. Sci..

[29] Yan Y., Huang S.-Y. (2019). Pushing the accuracy limit of shape complementarity for protein-protein docking. BMC Bioinform..

[30] Gabb H.A., Jackson R.M., Sternberg M.J.E. (1997). Modelling protein docking using shape complementarity, electrostatics and biochemical information. J. Mol. Biol..

[31] Verdonk M.L., Giangreco I., Hall R.J., Korb O., Mortenson P.N., Murray C.W. (2011). Docking Performance of Fragments and Druglike Compounds. J. Med. Chem..

[32] Chemical Computing Group ULC (2021). Molecular Operating Environment (MOE).

[33] Case D.A., Walker R.C., Cheatham T.E., Simmerling C., Roitberg A., Merz K.M., Luo R., Li P., Darden T., Sagui C. (2021). Amber 2021.

[34] Humphrey W., Dalke A., Schulten K. (1996). VMD: Visual molecular dynamics. J. Mol. Graph..

[35] Harvey M., Giupponi G., De Fabritiis G. (2009). ACEMD: Accelerating Biomolecular Dynamics in the Microsecond Time Scale. J. Chem. Theory Comput..

[36] Eastman P., Swails J., Chodera J.D., McGibbon R.T., Zhao Y., Beauchamp K.A., Wang L.-P., Simmonett A.C., Harrigan M.P., Stern C.D. (2017). OpenMM 7: Rapid development of high performance algorithms for molecular dynamics. PLoS Comput. Biol..

[37] Berman H.M., Westbrook J., Feng Z., Gilliland G., Bhat T.N., Weissig H., Shindyalov I.N., Bourne P.E. (2000). The Protein Data Bank. Nucleic Acids Res..

[38] Case D.A., Darden T., Cheatham T.E., Simmerling C., Wang J., Duke R.E., Luo R., Crowley M., Walker R., Zhang W. (2008). Amber 10.

