# Supplementary material for: Re-Exploring the Ability of Common Docking Programs to Correctly Reproduce the Binding Modes of Non-Covalent Inhibitors of SARS-CoV-2 Protease Mpro
Source: Pharmaceuticals (Basel). 2022 Jan 31;15(2):180. doi: 10.3390/ph15020180 (PMC8878732; doi:10.3390/ph15020180)
Supplement: Supplementary file 1 [file pharmaceuticals-15-00180-s001.zip › pharmaceuticals-1563251-suppl/Supplementary_material.pdf]

**Supplementary material**

**Table S1.**List of the M$^{pro}$ protein ligand-complexes used for this study. The name of the complex is composed by its PDB name and by the PDB code of the ligand small molecule crystallized within. For each system, it is also reported if the ligand is crystallized inside or outside the orthosteric binding pocket.

| Protein-ligand complex | Ligand IN/OUT of the catalytic pocket | Protein-ligand complex | Ligand IN/OUT of the catalytic pocket | Protein-ligand complex | Ligand IN/OUT of the catalytic pocket |
|---|---|---|---|---|---|
| 5R7Y-JFM | IN | 7AVD-S1W | IN | 5REA-JGP | OUT |
| 5R7Z-HWH | IN | 7AWU-S8B | IN | 5REC-T1J | OUT |
| 5R80-RZG | IN | 7B83-PK8 | IN | 5RED-JJG | OUT |
| 5R81-RZJ | IN | 7CA8-FNO | IN | 5REE-T1M | OUT |
| 5R82-RZS | IN | 7JU7-G65 | IN | 5REF-6SU | OUT |
| 5R83-K0G | IN | 7KX5-X7V | IN | 5REG-LWA | OUT |
| 5R84-GWS | IN | 7L0D-0EN | IN | 5REI-T1S | OUT |
| 5RE4-SZY | IN | 7L10-XEY | IN | 5RF0-T5D | OUT |
| 5RE9-LPZ | IN | 7L11-XF1 | IN | 5RF4-T5Y | OUT |
| 5REB-T0Y | IN | 7L12-XF4 | IN | 5RF5-HV2 | OUT |
| 5REH-AWP | IN | 7L13-XF7 | IN | 5RF8-SFY | OUT |
| 5REZ-T54 | IN | 7L14-XFD | IN | 5RF9-S7D | OUT |
| 5RF1-T5G | IN | 7L5D-XNJ | IN | 5RFA-JGY | OUT |
| 5RF2-HVB | IN | 7LDL-XV4 | IN | 5RFB-K3S | OUT |
| 5RF3-T5V | IN | 7LFE-XWS | IN | 5RFC-K1Y | OUT |
| 5RF6-NTG | IN | 7LMD-Y6A | IN | 5RFD-T6J | OUT |
| 5RF7-T67 | IN | 7LME-Y6J | IN | 5RGG-NZD | OUT |
| 5RFE-JGG | IN | 7LMF-Y6G | IN | 5RGJ-U0S | OUT |
| 5RG1-T9J | IN | 7LTJ-YD1 | IN | 5RGQ-U1V | OUT |
| 5RGH-U0M | IN | 7M8M-YSG | IN | 5RGS-S7V | OUT |
| 5RGI-U0P | IN | 7M8N-YSP | IN | 5RH4-UHG | OUT |
| 5RGK-U0V | IN | 7M8O-YSM | IN | 6YVF-A82 | OUT |
| 5RGU-UGD | IN | 7M8P-YSJ | IN | 7ABU-R6Q | OUT |
| 5RGV-UGG | IN | 7M8X-YTJ | IN | 7AF0-R9W | OUT |
| 5RGW-UGM | IN | 7M8Y-YTM | IN | 7AGA-LZE | OUT |
| 5RGX-UGP | IN | 7M8Z-YTV | IN | 7AMJ-RMZ | OUT |
| 5RGY-UGS | IN | 7M90-YTS | IN | 7AOL-RQH | OUT |
| 5RGZ-UH1 | IN | 7M91-YU4 | IN | 7AQI-QEL | OUT |
| 5RH0-UH4 | IN | 7MPB-ASC | IN | 7AWR-S7W | OUT |
| 5RH1-UGV | IN | 7N44-06I | IN | 7AWW-CLU | OUT |
| 5RH2-UH7 | IN | 7N8C-YD1 | IN | 7AXM-93J | OUT |
| 5RH3-UHA | IN | 7NT3-UQZ | IN | 7AXO-QCP | OUT |
| 5RH8-UHM | IN | 7NUK-USH | IN | 7KVL-X4P | OUT |
| 5RHD-US7 | IN | 7P51-5P9 | IN | 7KVR-X4V | OUT |
| 6M2N-3WL | IN | 7S3K-Z26 | IN | 7KW5-X4Y | OUT |
| 6W63-X77 | IN | 7S3S-860 | IN | 7LDX-R9V | OUT |
| 6W79-X77 | IN | 7S4B-87H | IN | 7LFP-XY4 | OUT |
| 7A1U-FUA | IN | 5RE5-T0J | OUT | | |
| 7ANS-RNW | IN | 5RE6-O0S | OUT | | |
| 7AP6-RQN | IN | 5RE7-T0S | OUT | | |
| 7AQE-RV5 | IN | 5RE8-T0V | OUT | | |

**Table S2.** Table showing the result of the solvent exposure analysis for each $M^{pro}$ crystallographic ligand. The pose considered for the calculation was the crystallographic one. The name of the complex is composed by its PDB name and from the PDB code of the ligand small molecule crystallized within. For each system, it is also reported if the ligand is located inside or outside the orthosteric binding pocket.

| Protein-ligand complex | Ligand IN/OUT of the catalytic pocket | Percentage of ligand surface area exposed to the solvent |
|---|---|---|
| 5R7Y-JFM | IN | 16,94 |
| 5R7Z-HWH | IN | 19,16 |
| 5R80-RZG | IN | 15,57 |
| 5R81-RZJ | IN | 18,94 |
| 5R82-RZS | IN | 19,11 |
| 5R83-K0G | IN | 8,99 |
| 5R84-GWS | IN | 7,18 |
| 5RE4-SZY | IN | 13,64 |
| 5RE9-LPZ | IN | 46,21 |
| 5REB-T0Y | IN | 19,78 |
| 5REH-AWP | IN | 51,67 |
| 5REZ-T54 | IN | 16,61 |
| 5RF1-T5G | IN | 18,89 |
| 5RF2-HVB | IN | 18,08 |
| 5RF3-T5V | IN | 13,80 |
| 5RF6-NTG | IN | 17,35 |
| 5RF7-T67 | IN | 10,46 |
| 5RFE-JGG | IN | 24,08 |
| 5RG1-T9J | IN | 13,87 |
| 5RGH-U0M | IN | 13,30 |
| 5RGI-U0P | IN | 12,83 |
| 5RGK-U0V | IN | 51,80 |
| 5RGU-UGD | IN | 8,71 |
| 5RGV-UGG | IN | 19,31 |
| 5RGW-UGM | IN | 9,91 |
| 5RGX-UGP | IN | 9,81 |
| 5RGY-UGS | IN | 35,21 |
| 5RGZ-UH1 | IN | 6,98 |
| 5RH0-UH4 | IN | 8,93 |
| 5RH1-UGV | IN | 11,18 |
| 5RH2-UH7 | IN | 12,30 |
| 5RH3-UHA | IN | 11,67 |
| 5RH8-UHM | IN | 12,74 |
| 5RHD-US7 | IN | 17,24 |
| 6M2N-3WL | IN | 3,69 |
| 6W63-X77 | IN | 15,01 |
| 6W79-X77 | IN | 17,64 |
| 7A1U-FUA | IN | 35,39 |
| 7ANS-RNW | IN | 10,39 |
| 7AP6-RQN | IN | 12,92 |
| 7AQE-RV5 | IN | 17,46 |
| 7AVD-S1W | IN | 34,92 |
| 7AWU-S8B | IN | 39,39 |
| 7B83-PK8 | IN | 27,91 |
| 7CA8-FNO | IN | 18,17 |
| 7JU7-G65 | IN | 22,89 |
| 7KX5-X7V | IN | 14,10 |
| 7L0D-0EN | IN | 13,87 |

| | | |
|---|---|---|
| 7L10-XEY | IN | 16,80 |
| 7L11-XF1 | IN | 13,15 |
| 7L12-XF4 | IN | 11,99 |
| 7L13-XF7 | IN | 16,68 |
| 7L14-XFD | IN | 13,15 |
| 7L5D-XNJ | IN | 24,72 |
| 7LDL-XV4 | IN | 18,35 |
| 7LFE-XWS | IN | 13,16 |
| 7LMD-Y6A | IN | 13,89 |
| 7LME-Y6J | IN | 14,81 |
| 7LMF-Y6G | IN | 13,35 |
| 7LTJ-YD1 | IN | 12,08 |
| 7M8M-YSG | IN | 12,68 |
| 7M8N-YSP | IN | 15,94 |
| 7M8O-YSM | IN | 16,77 |
| 7M8P-YSJ | IN | 15,81 |
| 7M8X-YTJ | IN | 14,90 |
| 7M8Y-YTM | IN | 17,74 |
| 7M8Z-YTV | IN | 15,02 |
| 7M90-YTS | IN | 15,90 |
| 7M91-YU4 | IN | 12,54 |
| 7MPB-ASC | IN | 18,89 |
| 7N44-06I | IN | 9,48 |
| 7N8C-YD1 | IN | 12,33 |
| 7NT3-UQZ | IN | 20,25 |
| 7NUK-USH | IN | 18,05 |
| 7P51-5P9 | IN | 15,67 |
| 7S3K-Z26 | IN | 18,78 |
| 7S3S-860 | IN | 15,57 |
| 7S4B-87H | IN | 15,62 |
| 5RE5-T0J | OUT | 49,62 |
| 5RE6-O0S | OUT | 48,87 |
| 5RE7-T0S | OUT | 38,26 |
| 5RE8-T0V | OUT | 45,35 |
| 5REA-JGP | OUT | 70,12 |
| 5REC-T1J | OUT | 51,69 |
| 5RED-JJG | OUT | 51,99 |
| 5REE-T1M | OUT | 33,11 |
| 5REF-6SU | OUT | 30,60 |
| 5REG-LWA | OUT | 38,49 |
| 5REI-T1S | OUT | 56,22 |
| 5RF0-T5D | OUT | 52,50 |
| 5RF4-T5Y | OUT | 44,20 |
| 5RF5-HV2 | OUT | 26,78 |
| 5RF8-SFY | OUT | 60,90 |
| 5RF9-S7D | OUT | 31,73 |
| 5RFA-JGY | OUT | 13,99 |
| 5RFB-K3S | OUT | 51,17 |
| 5RFC-K1Y | OUT | 49,15 |
| 5RFD-T6J | OUT | 49,80 |
| 5RGG-NZD | OUT | 49,33 |
| 5RGJ-U0S | OUT | 50,88 |
| 5RGQ-U1V | OUT | 2,48 |
| 5RGS-S7V | OUT | 31,97 |

| | | |
|---|---|---|
| 5RH4-UHG | OUT | 55,52 |
| 6YVF-A82 | OUT | 51,50 |
| 7ABU-R6Q | OUT | 59,53 |
| 7AF0-R9W | OUT | 76,15 |
| 7AGA-LZE | OUT | 28,37 |
| 7AMJ-RMZ | OUT | 61,11 |
| 7AOL-RQH | OUT | 54,82 |
| 7AQI-QEL | OUT | 58,98 |
| 7AWR-S7W | OUT | 48,45 |
| 7AWW-CLU | OUT | 53,95 |
| 7AXM-93J | OUT | 62,58 |
| 7AXO-QCP | OUT | 48,20 |
| 7KVL-X4P | OUT | 40,90 |
| 7KVR-X4V | OUT | 61,16 |
| 7KW5-X4Y | OUT | 34,07 |
| 7LDX-R9V | OUT | 29,89 |
| 7LFP-XY4 | OUT | 38,64 |

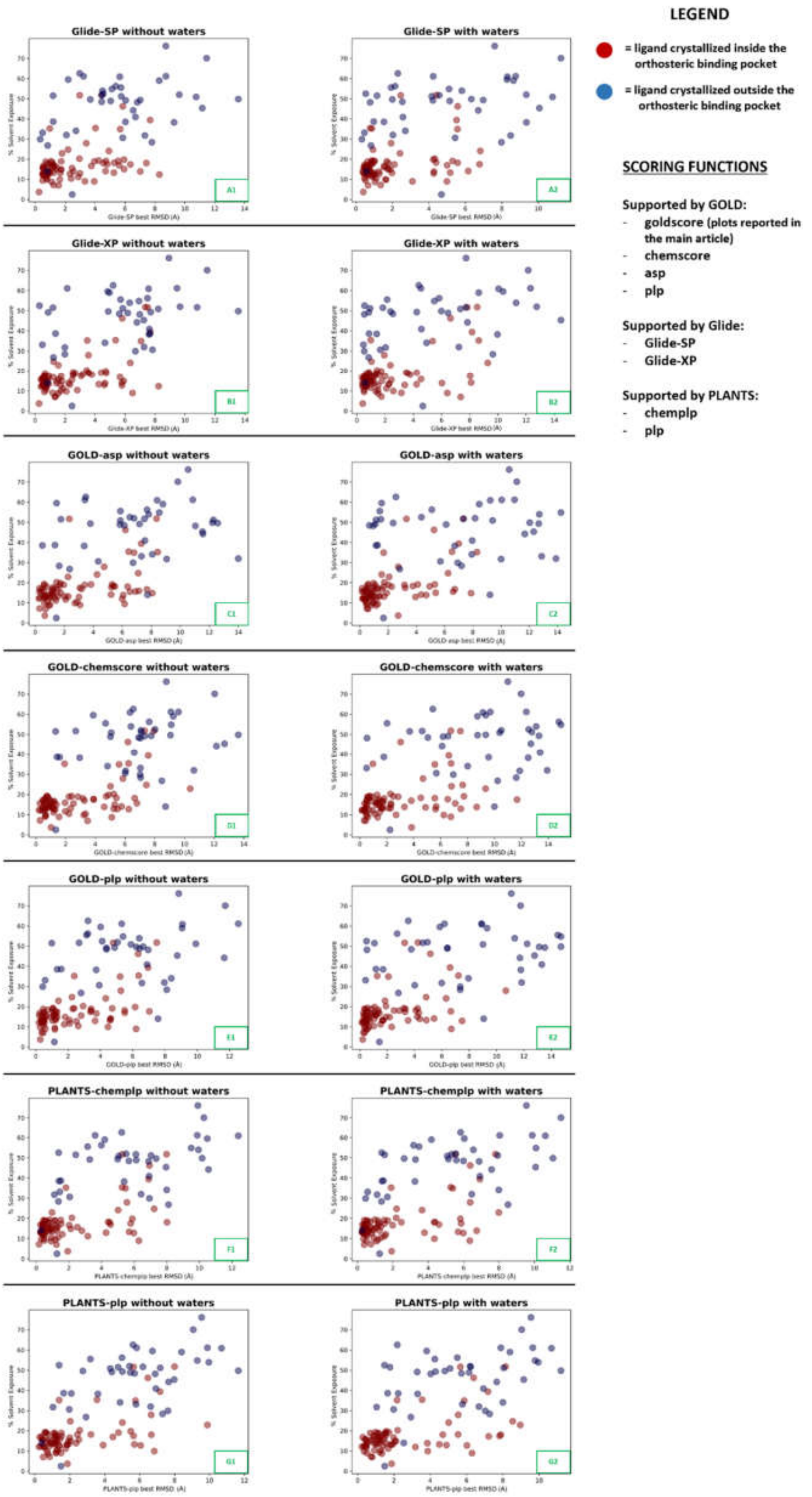


**Figure S1.**Scatter plots showing the different distribution of the RMSD values between the coordinates of the best pose from the different docking program-scoring function pairs (coming from the molecular docking experiments executed in this study) in function of the solvent exposure of the crystallographic ligand conformation. Each couple of plots (indicated with the same capital letter in green) represents the outcomes in the two "scenarios" considered, so all the graphs marked with number "1" correspond to the docking experiments in which the crystallographic water molecules were not considered in the calculation, while the plots with number "2" show the results for the case in which also water molecules 5 Å or nearer to the ligand are considered in the docking runs. In each plot, the x-axis represents the RMSD value between the coordinates of the best pose given by the docking experiment for a ligand in comparison with the coordinates of its crystallographic conformation. The y-axis represents the exposure to the solvent of the crystallographic ligand pose. The red dots represent the ligands that are originally crystallized inside the catalytic pocket, while the blue dots represent the ligands crystallized in the other parts of M$^{pro}$. As can be noticed, in all the plots the molecules showing the best values of RMSD are tendentially located inside the orthosteric pocket and characterized by a low solvent exposure of the original crystallographic pose.
